# Noncovalent Functionalization of Boron Nitride Nanotubes in Aqueous Media Opens Application Roads in Nanobiomedicine

**DOI:** 10.5772/60000

**Published:** 2014-01-01

**Authors:** Zhenghong Gao, Chunyi Zhi, Yoshio Bando, Dmitri Golberg, Takeshi Serizawa

**Affiliations:** 1 Laboratoire, Photonique Numérique et Nanosciences (LP2N), Institut d'Optique Graduate School & CNRS & Université de Bordeaux, Institut d'Optique d'Aquitaine (IOA), Rue François Mitterrand, Talence Cedex, France; 2 International Center for Materials Nanoarchitectonics (MANA), National Institute for Materials Science (NIMS), Tsukuba, Ibaraki, Japan; 3 Department of Organic and Polymeric Materials, Tokyo Institute of Technology, Ookayama, Meguro-ku, Tokyo, Japan

**Keywords:** Noncovalent functionalization, polymer, biomolecule, boron nitride nanotube, nanobiomedicine

## Abstract

Boron nitride nanotubes (BNNTs) are of intense scientific interest due to their unique physiochemical properties and prospective applications in various nanotechnologies, particularly nanobiomedicine. A critical problem hampering the application processing of BNNTs is the outer sidewall functionalization, which is primarily acquired to lead BNNTs dispersible in various solvents. Furthermore, the surface of BNNTs should be intelligently designed and precisely controlled to satisfy the specific demands of different applications. For these purposes, covalent and noncovalent approaches have been factually developed to help to extend the full potential of applications. Importantly, wrapping the outermost sidewall of BNNTs with either water-soluble polymers or biomolecules through weak noncovalent interactions has been proved to be efficient for giving BNNTs considerable dispersity in aqueous media, and endowing novel chemical functions to BNNTs with almost no change in their pristine physiochemical properties. This article summarizes recent progress in this field and addresses future perspectives on the noncovalent functionalization of BNNTs for promoting their application processing in various bio-related nanotechnologies.

## 1. Introduction

In recent years, one-dimensional (1-D) nanomaterials [1] such as nanotubes [2], nanowires [3], and nanorods [4] have attracted intensive scientific interest because of their unique nanoscale size and 1-D geometry-related physiochemical properties [5]. The properties of 1-D nanomateri-als are closely associated with their new quantum mechanical effect, size effect, and surface effect; they can be far different from those for conventional bulk materials [1]. In turn, the arising of 1-D nanomaterials holds great promises for developing novel nanosystems for many crucial technologies such as high-efficiency energy conversion devices [6] and environmentally friendly technologies [7]. Carbon nanotubes (CNTs) are the representatives of 1-D nanomaterials. The scientific interest shown to CNTs stems from their unique 1-D nanostructure and consequent multiple outstanding electronic, optic, thermal, and mechanic properties [8]. Structurally, this 1-D carbon allotrope is made by rolling up a two-dimensional (2-D) graphene sheet a one-carbon atom thick layer along a certain direction. The direction of the rolling up is theoretically represented by a pair of defined indices (*n, m*) [9]. The integer *n* and *m* denote the number of the unit vectors along two directions in the honeycomb crystal lattice of the graphene. The CNTs are classified into armchair (*n, n*), zigzag (*n, 0*), and chiral (*n, m*) properties, depending on the different roll-up directions [9]. On the other hand, CNTs can also be classified into single-walled CNTs (SWCNTs), double-walled CNTs (DWCNTs), and multi-walled CNTs (MWCNTs), according to the graphene sheet numbers. The properties of those CNTs are thus quite different from each other [10]. The tubular structure of CNTs was first clearly grown and observed twenty years ago [2], and the first report on this filament form of carbon materials was much earlier [11]. To date, the application of CNTs is still hampered by the pristine indispersity in most aqueous and organic solvents. In other words, as-produced CNTs tightly aggregate into bundles through the strong van der Waals interactions between the tubes' sidewalls [12]. At the same time, the electrical properties of SWCNTs are correlated with their specific chirality [13]. SWCNTs can thereby be metallic or semiconducting depending on the different chirality. Although several methods have been reported as being efficient for the isolation of CNTs with specific chirality, unfortunately, almost all those methods are currently limited to a batch scale. There are still very few efficient methods for the precise isolation of SWCNTs with specific chirality on a large scale to meet the needs of industry applications. In this sense, the functionalization of the sidewall of CNTs are primarily acquired not only to make them dispersible in aqueous and organic solvents, but also for extracting samples rich in single chiral tubes. Noncovalent [14] and covalent [15, 16] approaches have been explored to solve this problem.

**Figure 1.**
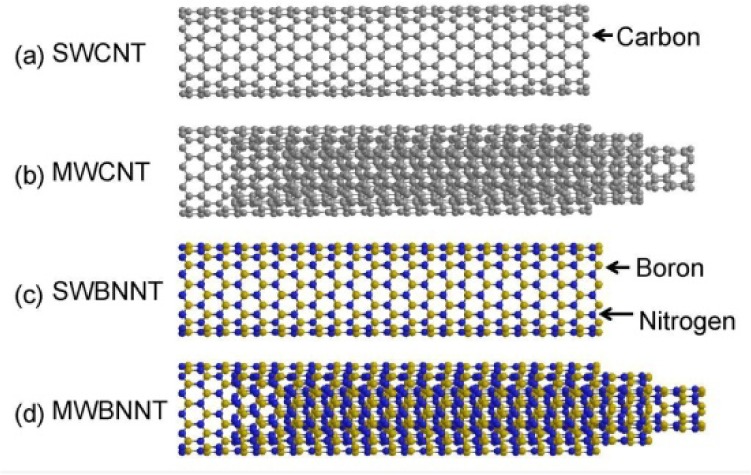
Molecular model of (a) SWCNT, (b) MWCNT, (c) SWBNNT and (d) MWBNNT

**Figure 2.**
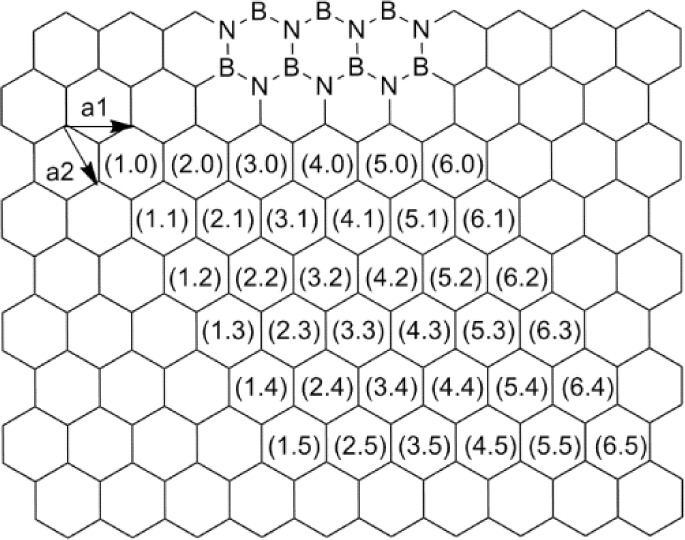
Vectors (n, m) for single-walled BNNT on an h-BN sheet

Boron nitride nanotubes (BNNTs) [17] are the representative inorganic analogues of CNTs (Figure 1) [18]. The theoretical prediction of BNNTs [19, 20] and their experimental synthesis [17] were successfully reported in 1994 and 1995, respectively. BNNTs are structurally similar to CNTs except that B and N atoms alternatively substitute C atoms in the graphene-like honeycomb crystal lattice, giving a one-atom thick hexagonal BN layer [21]. As shown in Figure 2, the same indices (*n, m*) are applicable for the definition of the chirality of these tubes as those for CNTs [22, 23]. However, in contrast to CNTs, BNNTs have an electric band-gap of around 5.5 eV independent of structural chirality and diameter [20, 24]. The distinct wide electric band-gap and high chemical stability in high temperature/oxidation environments [25] allow BNNTs invaluable for high-performance nanocomposite materials [26, 27] and biomedical applications such as drug delivery [28, 29]. Similar to CNTs, BNNTs have highly hydrophobic sidewalls and tightly aggregate together with their adjacent tubes via van der Waals forces [30], meaning that as-produced BNNTs exist as bundles or ropes. Therefore, BNNTs are un-dispersible in many aqueous and organic solvents [31]. The potential applications of BNNTs are hampered by their indispersity, similarly to those for CNTs. It is strongly required to clear this obstacle by exploiting new functionalization approaches by modifying the hydrophobic properties of BNNTs' sidewalls to make them primarily dispersible in many solvents, particularly in aqueous solutions [32]. The functionalizations of the sidewalls of CNTs with various synthetic polymers via covalent grafting or noncovalent wrapping have been widely investigated mostly due to the rapid progress in the synthesis methods. However, the corresponding studies on BNNTs lie far behind because of the serious lack of an efficient method for producing BNNTs of high quality and purity [32]. Several years ago, a key breakthrough for synthesizing BNNTs on a gram scale was achieved by a pioneer group in BNNTs research at the National Institute for Materials Science – NIMS in Japan [33-36]. Since then, research activities on BNNTs have started to grow very quickly. So far, many methods have been exploited including low temperature growth [37] and pattered formation on substrates [38]. However, the synthesis and post-purification of BNNTs are factually much more difficult in comparison to the same issues for CNTs [34]. Most samples currently involved in the research are still multi-walled BNNTs (MWBNNTs). High quality singlewalled BNNTs (SWBNNTs) samples are relatively rare in both the laboratory and commercial markets. Although the size and properties of SWBNNTs and MWBNNTs are different from each other, their basic structures of the outermost sidewalls are commonly the same. Therefore, the chemical functionalization approaches for modifying MWBNNTs should also be alternatives for SWBNNTs.

## 2. Overview of functionalization approaches for BNNTs

**Figure 3.**
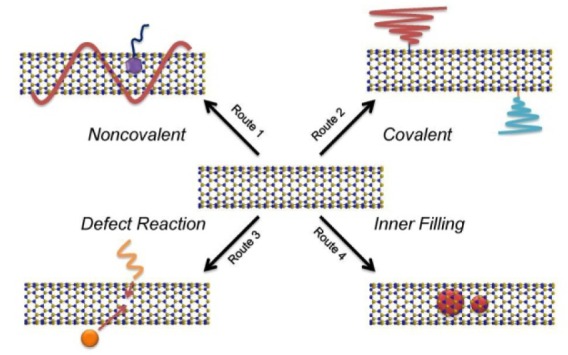
Functionalization approaches (routes) for BNNTs

For the sidewall functionalization of BNNTs, there are generally four main approaches available (Figure 3). The first functionalization approach is to covalently attach alien chemical groups to the -NH and/or -NH_2_ defect sites present on the sidewall of BNNTs, which are induced during the imperfect crystalline growth process or by chemical post-treatments such as harsh acid oxidation. Another covalent approach is to form chemical bonds by the use of boron or nitride sites based on boron or nitride chemistry (Figure 3). For example, Zhi et al. developed a covalent approach based on the reaction between the COCl group of stearoyl chloride and the amino groups (defect sites) on the BNNTs after refluxing the mixture for 120 h at 100 °C [31]. The resultant BNNTs were dispersible in organic solvents such as chloroform, *N, N*-dimethylacetamide, tetrahydofuran, *N, N*-dimethylformamide, acetone, toluene, and ethanol. Other covalent approaches for the functionalization of BNNTs include modification with amine-terminated poly(ethylene glycol) which forms ionic bonds with B sites on BNNTs sidewalls by heating 100 °C for several hours [30]; peeling the B-N bond by cyclic treating BNNTs with dimethyl sulfoxide (DMSO), in which DMSO molecules were added to the sidewalls of BNNTs through a nucleophilic attack of O on B and an electrophilic attack of S on N under the mixing processes of sonication and heating at 180 °C for 6 h [39]; fluorination by inducing F atoms during the stage of BNNTs growth, here F were covalently doped to N atoms due to their close electrone-gativities [40]; and interaction with Lewis bases under heating at 70 °C for 12 h and followed by sonicating for a few minutes, in which B on BNNTs surface behaved as Lewis acids and interacted with various Lewis bases [41]. The advantage of the covalent method is that the formed chemical bonds are quite stable and durable; the main drawbacks of a covalent approach are the changes of the pristine crystalline structure and properties, in particular, the destruction of the outer surface/sidewall structure, which can even lead to a poor biocompatibility.

On the other hand, the attachment of polymers and biomolecules onto BNNTs through noncovalent interactions such as π-π stacking interactions provides a novel and gentle approach for functionalization (Figure 3). For example, it has been demonstrated that a conjugated polymer of poly[*m*-phenylenevinylene-co-(2, 5-dioctoxy-*p*-phenylenevinylene)] (PmPV) is able to noncovalently wrap BNNTs [42]. PmPV-wrapped BNNTs were dispersible in chloroform, *N, N*-dimethylacetamide, and tetrahydofuran, but they were un-dispersible in water and ethanol. It can easily be seen that many approaches are workable for making BNNTs dispersible in organic solvents, but still un-dispersible in aqueous phases. However, the aqueous dispersion is preferable for most biological applications which are the major prospective of BNNTs, but only few efforts have been addressed to dispersing BNNTs in aqueous phases. For example, Zhi et al. reported the dispersion of BNNTs in water using a single-strand DNA wrapping approach via π-π stacking interactions [43]. Chen et al. reported the functionalization of BNNTs using an amphipathic dendritic structure comprising synthetic carbohydrate ligands at the chain ends that enable specific binding to receptors in solution. In this case, a pyrene group at the dendrimer focal point allows these dendrimers to be able to interact with the isoelectronic BNNT surface through π-π stacking and hydrophobic interactions. The simple noncovalent absorption of amphipathic dendritic structures enables the BNNTs surface to display the glycodendrimers capable of interacting with proteins and cells. The method should facilitate the applications of BNNTs in biosensing and bioimaging [44]. Wang et al. reported the aqueous noncovalent functionalization of BNNTs using an anionic perylene derivative, namely perylene-3, 4, 9, 10-tetracarboxylic acids tetrapotassium salts (PTAS) [45]. The interactions were the π-π stacking interactions between PTAS and BNNTs [46]. Plenty of surface-attached COOH groups gave dispersity and chemical activity to BNNTs in turn. PTAS-functionalized BNNTs can superiorly bind to a large variety of metal ions. Importantly, by employing a high temperature vacuum, heating the BNNTs to 1180 °C at a rate of 2 °C/min and continuous isothermal annealing the samples at the same temperature over 2 h, PTAS-BNNTs were converted into C-doping BNNTs which exhibited considerable semiconducting properties. In addition, the filling of the inner tube channel with small molecules [47] or inorganic nanoparticles [48-51] is another approach for functionalizing BNNTs (Figure 3).

To sum up the above-mentioned advancements in the dispersion and functionalization of BNNTs, it can be said that although notable covalent and noncovalent approaches have been developed, many new approaches are still highly desired for extending the processabilities of various technical applications.

## 3. Noncovalent approaches for dispersion and functionalization of BNNTs

Among the various approaches for the surface functionalization of both CNTs and BNNTs, the noncovalent attachment of polymers [52, 53] or small molecules [54, 55] based on weak inter-molecular interactions, such as π-π stacking [55], cation-π interactions [56], and hydrogen bonds [57], the preservation of pristine electric and optical properties, which are essential for biological applications, has been achieved [14]. On the other hand, due to the diversity of polymers and small molecules, different alien chemical groups can be intensively attached to the sidewall of NTs to endow different chemical functions benefiting many nanobio-related applications. Moreover, it is possible to remove noncovalently attached molecules from the sidewalls by suitable methods with minimum interruption to the intrinsic properties, which allow the recovery of the nanotube surface [14]. In addition, noncovalent polymer wrapping is efficient for the removal of the catalysis species, purification [58] and selective dispersion [59] of nanotube samples via a simple dispersion and extraction processes. In this sense, the development of approaches for the noncovalent functionalization of BNNTs is increasing scientific interest.

### 3.1 Aim and prospects

The unique 1-D geometry and outstanding chemical stability of BNNTs has promoted them as promising candidates for composite materials [27, 60-63] (mainly insulated thermal conducting polymeric composite) and nanobio-technological applications [44, 64], such as drug delivery systems (DDS) [29], magnetic resonance imaging (MRI) contrast agents [65], boron neutron capture therapy [66], and cancer cell treatments [67]. The strong van der Waals interactions between BNNTs makes them exist as bundles in their as-produced state. This results in the pristine indispersity of BNNTs in common aqueous and organic solvents [32]. However, for many applications, particularly bio-related fields, dispersion in water or a physiological buffer is preferred. On the other hand, new biological functions have been acquired for BNNTs to increase their availability in biological fields. Furthermore, the biocompatibility of BNNTs needs to be improved in most cases of biological applications [68]. The noncovalent approaches in aqueous solutions are efficient for functionalizing the sidewalls of BNNTs to address a broad range of applications, particularly opening many roads in nanobio-medicine. In this context, the contribution is made to provide a comprehensive understanding on the subject of the noncovalent functionalization of BNNTs with either synthetic polymers or biomolecules in aqueous phases.

### 3.2 Noncovalent functionalization of BNNTs with synthetic polymers

**Figure 4.**
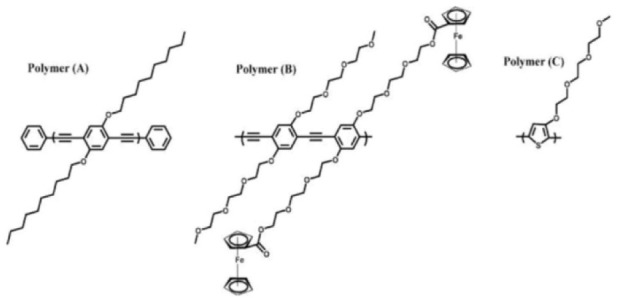
Chemical structures of the PPE derivatives (Polymers A and B) and the regioregular head-to-tail polythiophene derivative (Polymer C). (Reproduced with permission from ref 70. Copyright (2010) American Chemical Society).

**Figure 5.**
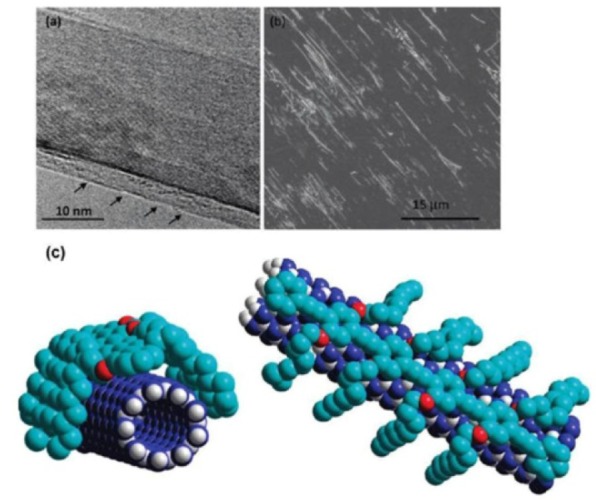
(a) HR-TEM image at the BNNT sidewalls functionalized with the PPE derivative (Polymer A). A layer of the PPE derivative can be clearly seen as indicated by arrows. (b) The SEM image of the aligned BNNTs functionalized with Polymer A. (c) Schematic models of composite material of BNNTs and Polymer A at various viewing angles. (Reproduced with permission from ref 70. Copyright (2010) American Chemical Society).

Synthetic polymers containing aromatic subunits can interact with the sidewall of BNNTs via π-π stacking interactions [69]. The earliest report on wrapping BNNTs with a π-conjugate conducting polymer-PmPV was produced by Zhi et al. [42]. The functionalization was conducted by simply sonicating the mixture of BNNTs with PmPV. A homogeneous dispersion was obtained after removing insoluble materials via a centrifugation process. The resultant PmPV-wrapped BNNTs were dispersible in organic solvents, such as chloroform, *N, N*-dimethylacetamide, tetrahydrofuran, etc., whereas they were un-dispersible in water, ethanol, etc. Velayudham et al. investigated the noncovalent functionalization of BNNTs using three conjugated poly(*p*-phenylene ethynylene)s (PPEs) (Polymers A and B) and polythiophene (Polymer C) derivatives through the strong π-π stacking interactions between the polymers and BNNTs [70]. The chemical structures of these polymers are shown in Figure 4. The resultant BNNTs are dispersible in organic solvents such as chloroform, methylene chloride, and tetrahydrofuran. A layer of the PPE derivative can be clearly seen on the surface of BNNTs in the high-resolution transmission electron microscopic (HR-TEM) image (Figure 5). The absorption and emission of the PPE derivatives attaching to BNNTs showed red shifts in comparison with free PPEs due to the enhanced planarization, while the polythiophene derivative showed blue shifts by the strong disruption of its π plane.

**Figure 6.**
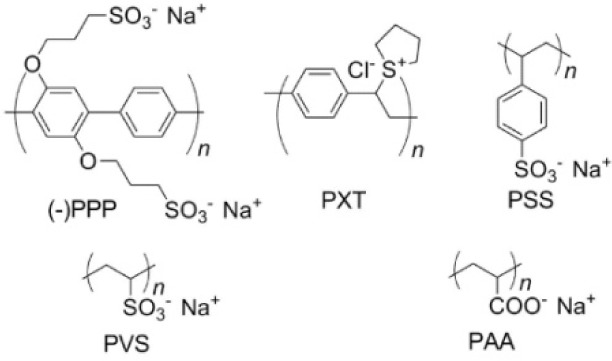
Chemical structures of water-soluble several synthetic polymers. (Reproduced with permission from ref 72. Copyright (2012) Nature Publishing Group).

**Figure 7.**
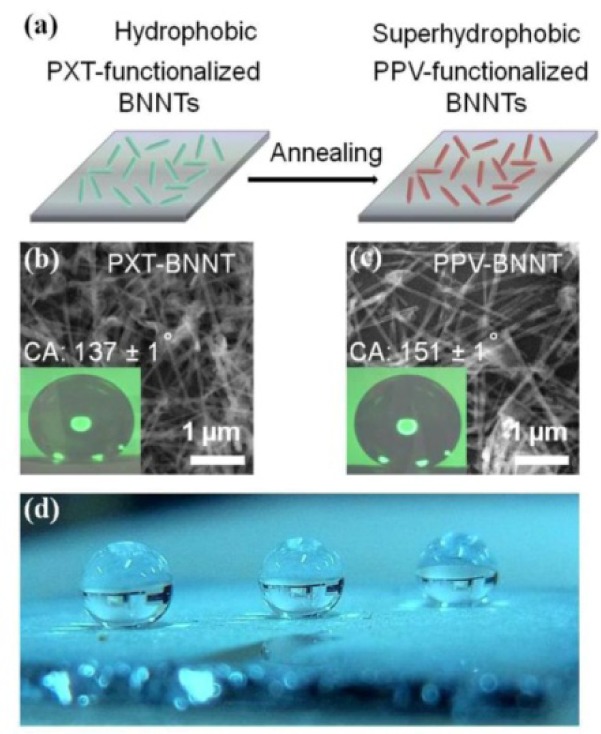
(a) Schematic image of the conversion of PXT into PPV on the BNNT sidewalls via thermal treatment, Scanning electron microscopy (SEM) image of the film surfaces coated with (b) PXT- and (c) PPV-functionalized BNNTs coated on a Si wafer, and (d) an optical photo of water droplets on superhydrophobic PPV-functionalized BNNTs. The insets in (b) and (c) show water droplets used for contact angle measurements. (Reproduced with permission from ref 72. Copyright (2013) Nature Publishing Group).

Even though it has been shown that several examples of polymers can function as dispersants for functionalizing the sidewall of BNNTs, details of the interactions of different polymers with BNNTs have not yet been fully understood [71]. Our group has recently investigated the potential of different water-soluble polymers, including a poly(*p*-phenylene) derivative ((-)PPP), poly(xylydiene tetrahydrothiophene) (PXT), poly(sodium styrene sulfonate) (PSS), poly(sodium vinyl sulfonate) (PVS), and poly(sodium acrylate) (PAA), for dispersing BNNTs in water (Figure 6) [72]. Our investigations have shown that (-)PPP has the highest potential for dispersing BNNTs among these polymers. These results indicated that polymers with an extensive π-plane show stronger interactions with BNNTs than those with a smaller π-plane, suggesting that the π-π stacking interactions play a very important role in the functionalization of BNNTs with polymers. Importantly, through a simple chemical conversion of PXT to PPV, we have obtained a film composed of PPV-functionalized BNNTs. Contact angle (CA) measurements suggested that this film exhibited a CA of 151 ± 1° which allows it to be non-wetting/superhydrophobic (Figure 7). This finding is promising for the construction of materials with non-fouling bio-surfaces. A molecular dynamic (MD) simulation study on the interfacial binding interactions of BNNTs and various polymers including PmPV, polystyrene (PS), and polythiophene (PT) using a density functional theory (DFT) was carried out by Nasrabadi et al. in 2010 [71]. Computer calculations of dihedral angle (θ) were conducted to determine the interaction energy between BNNTs and polymer molecules, and the morphology of polymers stacked onto the BNNT surface. The results showed that the specific monomer structure of the polymer and BNNT radius had a strong influence on the interaction strength, but the influence of temperature is likely negligible. Among PmPV, PS and PT, the order for stronger binding was PT, PmPV and PS. Moreover, they have revealed that the BNNT-polymer interactions are much stronger that those of the similar CNT-polymer composites, supporting the idea that those BNNTs are excellent candidates for the construction of high-performance polymer composite materials and bio-coatings.

### 3.3 Noncovalent functionalization of BNNTs with biomolecules

Even though many synthetic polymers are available for functionalizing BNNTs sidewalls, most synthetic polymers are supplied by organic and polymer synthesis, which is sometimes complicated. Another problem is that the biocompatibilities of synthetic polymers are still almost secrets. In this regard, water-soluble biomolecules offer great opportunities for the functionalization of BNNT due to their diversity of structures and functions [73]. They are low-cost, biocompatible, and require no precise synthesis. Biomolecules refer to chemical compounds found in living organisms. The most prominent classes of biomolecules are peptides, proteins, DNAs, RNAs, saccharides and lipids. Biomolecules often exhibit specific complementary interactions such as antibody-antigen, hormone-receptor, and protein-protein/nucleic, acid-nucleic, acid/nucleic, and acid-protein interactions. In combination with the important characteristics of biomolecules, self-assembly and biological evolution, the functionalization of nanomaterials with biomolecules appears as an important research area of materials chemistry [74]. It has been well proven that many biomolecules can be employed as dispersants and/or functional reagents for dispersing and functionalizing CNTs [74]. With a consideration of the similarity in the structure, many biomolecules can be similarly efficient for dispersing and functionalizing BNNTs. Research on this topic has just started to draw full attention recently.

Taking the many advantages of biomolecules into account, the dispersing and functionalizing BNNTs in an aqueous solution were performed using peptides [75], flavin mononucleotide (FMN) [76], DNA [43] / nucleotides [77] and polysaccharides [78]. These studies were firstly aimed at making BNNTs well dispersible in an aqueous solution via the functionalization of biomolecules, thereby opening a new pathway for nanobio-related applications in medicine. The second objective of these studies was to endow new physiochemical properties to BNNTs after functionalizing with biomolecules. An additional objective is to assemble quantum dots (QDs) and proteins onto biomolecules-functionalized BNNTs surfaces to form novel hybrids with unique optical properties applicable in bioimaging. The functionalization of BNNTs using water-soluble biomolecules not only endows a good dispersity to BNNTs, but also offers new physiochemical functions to BNNTs and opens applications roads in nanobiomedicine.

#### 3.3.1 Noncovalent functionalization of BNNTs using artificial peptides

A good aqueous dispersion of BNNTs has primarily been acquired for biological applications. Peptides have diverse functions due to the diversity of amino acids contained in the sequence of a certain peptide as well as their facilitated conjugating approach. Researchers have suggested that peptides can interact with the hydrophobic sidewall of CNTs via π-π stacking or hydrophobic interactions [79], which results in the individualization of CNTs. In consideration of the similarity between the crystalline structures of BNNTs and CNTs, it was reasonably anticipated that peptides were able to interact with BNNTs in a similar fashion as CNTs. We have artificially synthesized an aromatic CNTs binding peptide, denoted as B3 (HWSAW-WIRSNQS), and anticipated for its interaction with BNNTs via π-π stacking and/or hydrophobic interactions [75].

**Figure 8.**
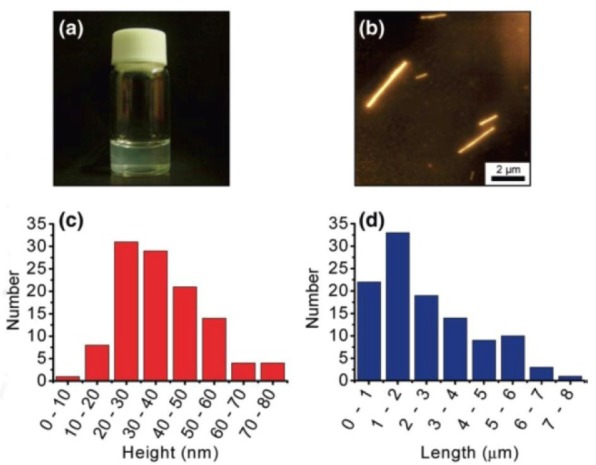
(a) Dispersion of B3-BNNT complexes, (b) AFM image of the complexes, and statistical AFM (c) height and (d) length analyses of the complexes collected from 50 different images (the total number of BNNTs was 112). (Reproduced with permission from ref 75. Copyright (2010) American Chemical Society).

In a typical experiment, BNNTs and a peptide solution were mixed, followed by a mild sonication for dispersing, the insoluble materials were removed by a centrifugation procedure, then the supernatant above 70–80 % of the dispersion was collected for further characterizations (Figure 8). The AFM characterizations on the morphologies suggested that B3-functionalized BNNTs could be excellently dispersed on the mica surface by a slow spin-coat process. The investigations on the conformation suggested that B3 adopted an α-helical conformation in an aqueous solution. B3 showed a considerable dispersing ability for BNNTs. The spectroscopic studies indicated that the π-π interactions existed between B3 and BNNT. The peptide-functionalized BNNTs have promising applications in electronics, optical devices, and biological fields due to their excellent dispersion and unique physical properties.

#### 3.3.2 Noncovalent functionalization of BNNTs using DNA and application for liquid crystal

**Figure 9.**
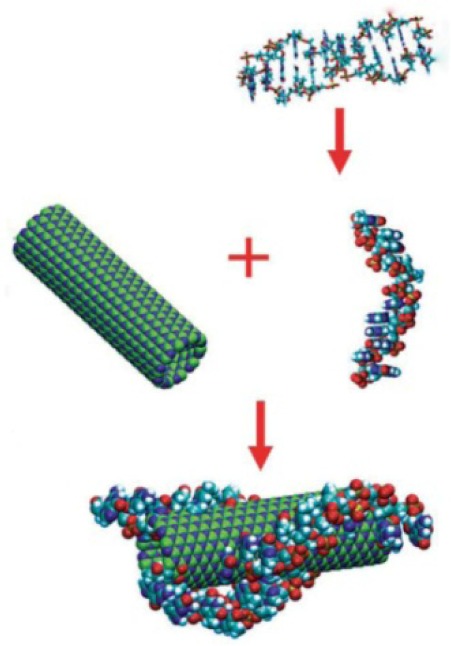
The process of the fabrication of the DNA-BNNT hybrid. (Reproduced with permission from ref 43. Copyright (2007) John Wily and Son).

In materials science, the high-concentration dispersion of BNNTs is acquired for increasing their processability. In particular, it offers the opportunity to understand the phase behaviour in solvents. DNA is a central biomolecule with many amazing properties. The aromatic nucleobases within DNA structures are capable of interacting strongly with the sidewalls of BNNT via π-π stacking and/or hydrophobic interactions (Figure 9). Zhi et al. dispersed BNNTs in an aqueous solution containing single-strand DNA (ACG TAC GTA ACG TAC GTA CGT ACG TAC) to obtain a sufficiently high-concentration dispersion [43]. The nematic liquid crystal state of BNNTs was observed from this aqueous high-concentration BNNTs dispersion based upon a simple filtration process. Clearly, an amorphous layer of about 5–20 nm was observed in the TEM image, suggesting the un-uniform attachment of DNA to the surface of BNNTs. A thermogravimetic analysis (TGA) suggested that 20 wt% DNA were attached to the BNNTs surface. Under optimized experimental conditions, a dispersion containing 0.2 wt% BNNTs could be obtained using a single-strand DNA. Apparent shifts were identified in both absorption and emission spectra of BNNTs and DNA, suggesting the presence of strong π-π stacking interactions of BNNT and DNA.

**Figure 10.**
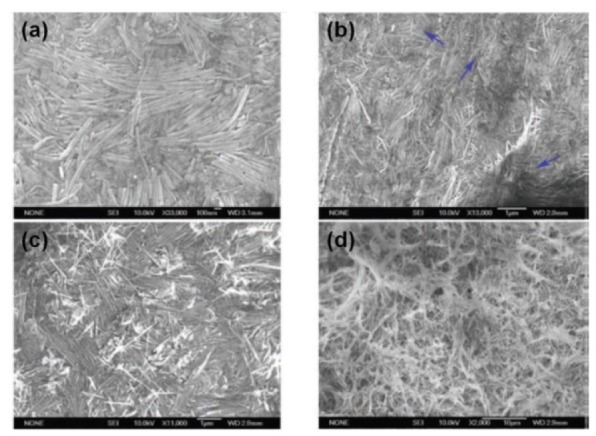
(a, b) Nematic ordering of BNNT ensembles (the singularities as shown with arrows in b)); (c) SEM images of the ensembles when a 0.1 wt% BNNTs solution was used for filtering (the BNNTs are less ordered); (d) as the BNNT concentration decreases, the morphology of the DNA–BNNT hybrid mat is affected by the water flow, and no BNNT ordering takes place. (Reproduced with permission from ref 43. (2007) John Wily and Son).

The assembly and phase behaviour of BNNTs in both solution and bulk states are fundamental for understanding the performance of materials, particularly for liquid crystal. In the aforementioned paper [43] a BNNTs mat was obtained by treating the filtered mixture of BNNT-DNA at 700 °C for 30 min. The SEM images showed that the mat contains a nematic order phase (Figure 10). The ordering behaviour depended on the concentration of BNNTs and the filtering process. The functionalization of BNNTs with DNAs may be useful for the development of novel biodevices.

#### 3.3.3 Noncovalent functionalization of BNNTs using Flavin Mononucleotide (FMN) and application for visible-light emission

Biomolecules, such as peptides and DNA, are able to interact with BNNTs. However, the precise interactions between these biomolecules and BNNTs are very complicated, and are hard to fully understand at the present stage. This difficulty has led us to the idea that small aromatic biomolecules are probably workable on functionalizing BNNTs. Those biomolecules containing aromatic moieties provide simple models for understanding the interactions. Furthermore, unlike macromolecules, small molecules can fully cover the surface of BNNTs, allowing the perfect isolation of BNNTs from their surroundings which is a key point for sustaining their optical properties. FMN is the phosphorylated derivative of vitamin B2. Its molecular structure consists of an aromatic isoalloxazine ring and a phosphate moiety. FMN is anticipated to interact with the hydrophobic surfaces of BNNTs with its isoalloxazine ring via π-π stacking interactions [76]. This establishes a noncovalent sidewall chemical pathway not only for dispersing and functionalizing BNNTs in an aqueous solution, but also for further integrating these innovative BNNTs-based hybrids into new composite materials and devices.

**Figure 11.**
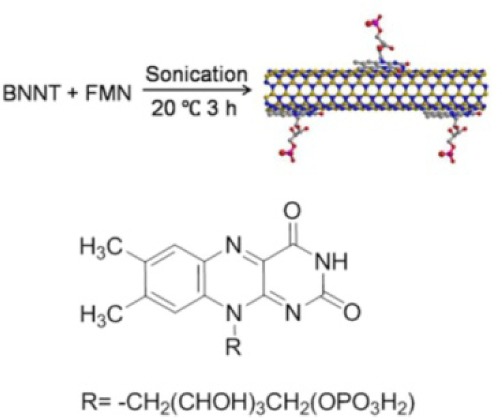
The representation of the formation process of a FMN/BNNT nanohybrid. (Reproduced with permission from ref 76. Copyright (2011) American Chemical Society).

**Figure 12.**
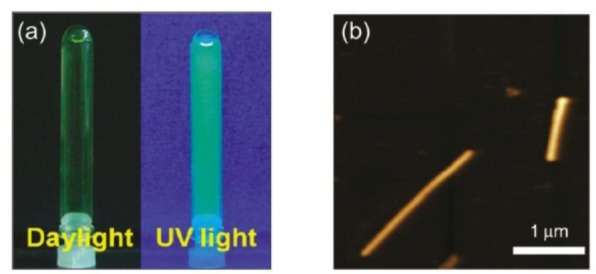
(a) The dispersion of BNNTs in the aqueous FMN solution under daylight and UV light and (b) the typical AFM image of FMN/BNNT nanohybrid. (Reproduced with permission from ref 76. Copyright (2011) American Chemical Society).

After dispersing the mixture of BNNTs with FMN using a sonicator under a mild condition, the disentanglement of BNNTs in water was achieved and thus FMN-functionalized BNNTs were obtained, denoted as FMN-BNNT nanohybrids (Figure 11). The spectroscopic results suggested the presence of strong π-π interactions between FMN and BNNTs. Optical measurements suggested that FMN-BNNT nanohybrids emitted a bright green fluorescence (Figure 12). Further studies suggested that the fluorescent intensities were stable in wide pH and temperature ranges. These nanohybrids are valuable for use as promising visible-light emitters that can be used as nanoscale biomarkers or biosensors for imaging or detecting local environments in biological systems.

#### 3.3.4 Noncovalent functionalization of BNNTs using nucleotides and application for Quantum Dots (QDs) decoration

**Figure 13.**
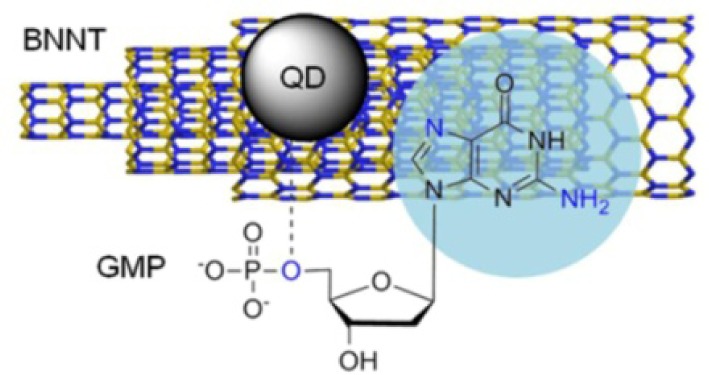
Representation of the decoration of BNNT sidewalls with CdS QDs assisted by GMP. (Reproduced with permission from ref 75. Copyright (2011) Royal Society of Chemistry).

The fluorescence of pristine BNNTs is located in the deep-UV range, which hampers the applications of BNNTs in bio-related fields. Nucleotides as the main chemical compounds of DNA and RNA are central molecules in biochemistry, and have structural properties similar to FMN. The idea is that nucleotides can interact with BNNTs via π-π stacking interactions [77]. Meanwhile, a previous piece of research has shown that guanosine 5′-monophosphate (GMP) caps cadmium sulfide (CdS) QDs via N7 of pyrimidine and/or -NH_2_ of purine, and P-O-5′-sugar, producing GMP-capped CdS QDs [80]. In this sense, free π-electrons should be presented on the GMP-capped QD surfaces. Those π-electrons should be capable of interacting with BNNT sidewalls. Thus, the decoration of BNNTs with GMP-capped CdS QDs is considered as a valuable way to further functionalize the BNNTs (Figure 13). Because these CdS QDs have fluorescence in the visible-light range at longer wavelengths than BNNTs, new fluorescence can be endowed to BNNTs after CdS QDs' decoration.

**Figure 14.**
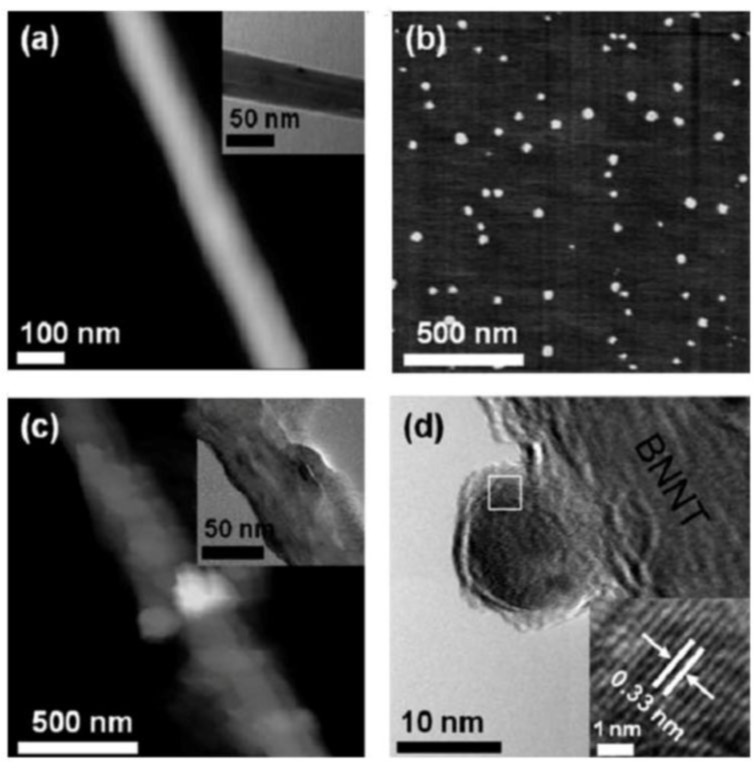
Representation of the decoration of BNNT sidewalls with CdS QDs assisted by GMP. (a) High-resolution AFM image of a GMP-modified BNNT. The inset shows the corresponding TEM image. (b) AFM image of GMP capped CdS QDs. (c) High-resolution AFM image of CdS/GMP@BNNT hybrids. The inset shows the corresponding TEM image. (d) Representative HR-TEM image of the hybrids. The inset shows the enlarged square portion. (Reproduced with permission from ref 75. Copyright (2011) Royal Society of Chemistry).

The nucleotides used included adenosine 5′-monophosphate (AMP), adenosine 5′-diphosphate (ADP), adenosine 5′-triphosphate (ATP), GMP, guanosine 5′-diphosphate (GDP), guanosine 5′-triphosphate (GTP), uridine 5′-monophosphate (UMP), cytidine 5′-monophosphate (CMP), and guanosine (Gua). The potentials of nucleotides for dispersing were compared quantitatively. The results indicated that the order of mononucleotides for better BNNTs dispersion was GMP > AMP ∼ UMP > CMP. The monophosphates were much better than the corresponding di- and triphosphates. HR-TEM images showed that the decoration of BNNTs with CdS QDs was achieved by using GMP as a linking reagent (Figure 14), which enabled the largest amounts of BNNTs to disperse in aqueous solution among nucleotides. Optical characterizations suggested that CdS/GMP@BNNT hybrids hold a new fluorescence in the visible-light range. The investigations of the interactions between nucleotides and BNNTs were helpful for understanding the chemistry of nucleotide-inorganic interfaces, which are extremely important for developing bio-nanotechnologies. New fluorescence in the visible-light range was endowed to BNNTs after successful decoration with CdS QDs. This will be beneficial in extending the utilization of BNNTs for bio-related applications.

#### 3.3.5 Noncovalent functionalization of BNNTs using natural polysaccharides and application for proteins decoration

**Figure 15.**
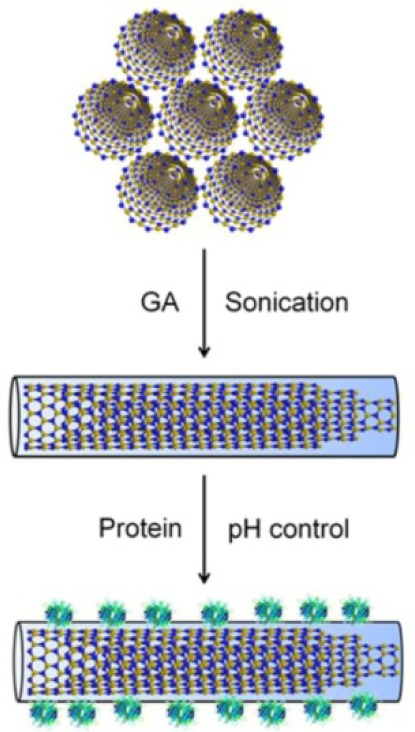
Schematic representation of disentanglement of BNNTs via functionalization with GA for proteins immobilization (Reproduced with permission from ref 78. Copyright (2012) Royal Society of Chemistry).

**Figure 16.**
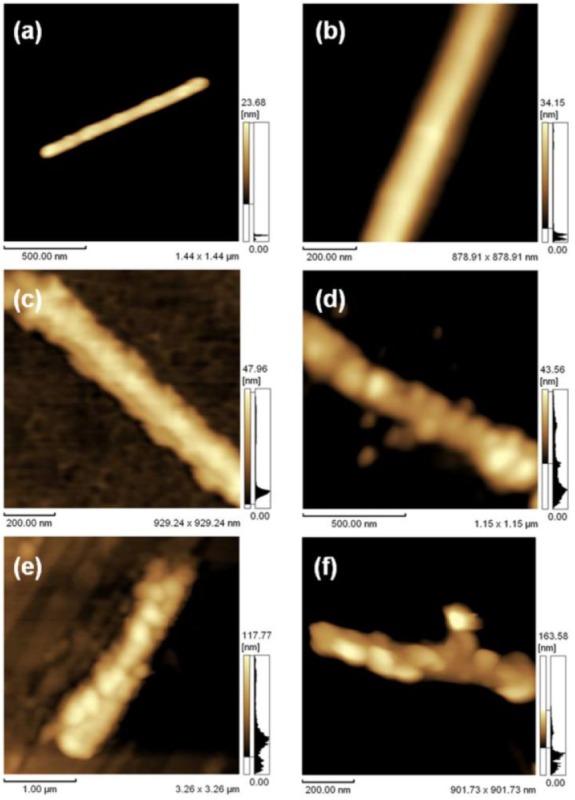
(a, b) AFM images of GA-functionalized BNNTs; (c) SAv (d) BSA (d) Lyz and (e) IgG on GA-functionalized BNNTs. (Reproduced with permission from ref 78. Copyright (2012) Royal Society of Chemistry).

The previous investigations showed that peptides, DNA, FMN, and nucleotides were able to work on the sidewall functionalization of BNNTs. However, these molecules are frequently produced by precise synthesis. To promote the prospective applications, new approaches for sidewall functionalization with lower-cost, better efficiency, and biocompatibility are in high demand. In this sense, natural polysaccharides are excellent candidates due to their cheapness and biocompatibility. Among them, gum arabic (GA) is an abundant polysaccharide in nature with highly branched complex molecular properties. GA is also highly water-soluble and easily chemical post-functionalization. Just recently, the use of GA has started to be extended into nanoscience and nanotechnology. We have shown that GA can be used to disperse and functionalize BNNTs in an aqueous solution [78]. The disentanglement of BNNT from raw bundled materials was achieved by functionalizing them with GA. These GA-functionalized BNNTs offered an opportunity for studying the physiochemical properties of single disentangled BNNTs. Afterwards, to show the advantage of GA-functionalized BNNTs, several functional proteins (streptavidin (SAv), lysozyme (Lyz), bovine serum albumin (BSA), and immunoglobulin G (IgG)) were successfully decorated onto the GA-functionalized BNNT surfaces by using the electrostatic interactions between proteins and GA-functionalized BNNTs (Figure 15, 16). The successful assembly of proteins on the surface of GA-functionalized BNNTs shows an important initial step for developing BNNT-based bio-devices.

**Figure 17.**
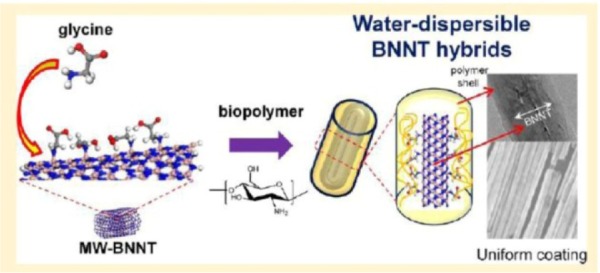
The schematic of the fabrication of the polysaccharide-BNNT hybrids. (Reproduced with permission from ref 81. Copyright (2013) American Chemical Society).

We have developed a strategy to improve the interfaces between polymers and BNNTs by incorporating glycine (NH_2_-CH_2_-COOH, Gly) in between as a bifunctional reagent: the amine group of Gly binds to B sites of BNNTs, and the carboxylic group offers functional sites for anchoring with polyelectrolytes (Figure 17). Gly-functionalized BNNTs also stabilize disentangled nanoobjects in aqueous suspension, and strongly absorb polysaccharides via electrostatic interactions between Gly-BNNTs and polymers, such as polysaccharides, polyanions, and polyzwitterionic polymers [81]. The formation of stable and uniform polymer-BNNTs nanometric complexes provides unique nanoplatforms which may be useful as vehicles for drug delivery and scaffolds for tissue engineering.

#### 3.3.6 Noncovalent functionalization of BNNTs using lipids and application for length cutting

**Figure 18.**
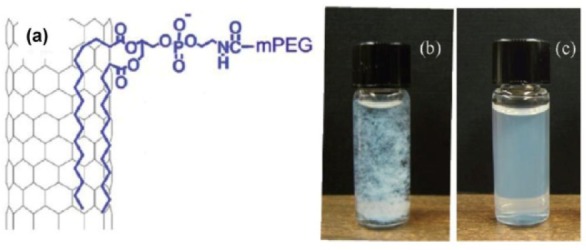
(a) Schematic representative of a BNNT functionalized by an mPEG-DSPE molecule. (b) The extracted BNNT bundles in ethanol. (c) Well-dispered mPEG-DSPE/BNNTs in water (after 2 h of sonication). (Reproduced with permission from ref 83. Copyright (2012) American Chemical Society).

The great promise of BNNTs for many biomedical applications has recently been fulfilled. For example, BNNTs offer a high potential for use in boron neutron capture therapy [66] due to the high neuron catching ability of boron. However, an aqueous dispersion of BNNTs must be achieved prior to any biological experiments. In the meantime, CNTs with short lengths are more biocompatible and have less toxicity in the tested cells and in animals than long ones [82]. The original lengths of as-produced BNNTs can be up to several tens of μm, which is too long for testing in most biological samples, particularly for their internalization into living cells [68]. The toxicity of BNNTs on human embryonic kidney (HEK293) cells has recently been found [68]. The origin of the toxicity is probably due to the large axial size. It is therefore necessary to develop a procedure for cutting and controlling the lengths. Very recently, Lee et al. have functionalized BNNTs with a PEGylated phospholipid [methoxy-poly(ethylene glycol)-1, 2-distearoyl-sn-glycero-3-phosphoethanolamine-N conjugates (mPEG-DSPE)] (Figure 18) [83]. The fatty acid chains of mPEG-DSPE can strongly bind around the sidewall of BNNTs through van der Waals, charge transfer, and hydrophobic interactions, whereas the mPEG chain interacts with water molecules through hydrogen bonds, thereby leading to mPEG-functionalized BNNTs being greatly dispersible in water. No notable precipitations were found in the water-dispersion of BNNTs containing mPEG-DSPE after being maintained up to three months. This is a great improvement in the stability of dispersed BNNTs in water. The hydrogen bonds between water molecules and the hydrophilic tail of mPEG are found to be essential for keeping the dispersion of the BNNTs stable. Furthermore, the length of the distribution can be controlled within the range of 500 nm by simply prolonging the sonication time up to 20 h. This procedure is easy to control and scale up. Although the exact function of mPEG molecules in the cutting process needs to be understood more carefully. This finding is still very useful for integrating BNNTs into valuable biological systems.

#### 3.3.7 In vitro and in vivo evaluation of noncovalent functionalization of BNNTs

**Figure 19.**
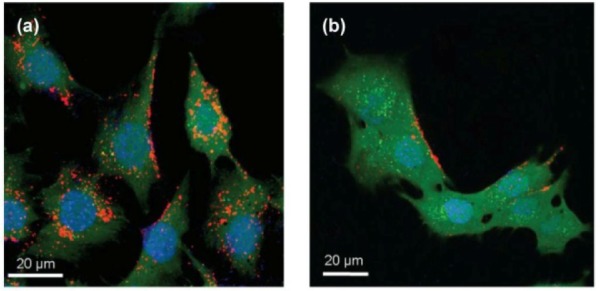
Confocal fluorescent imaging of QDs-labeled PLL-functionalized BNNTs internalized and taken up by C2C12 cells (a), and the inhibition after treatment with sodium azide (b). (Reproduced with permission from ref 84. Copyright (2010) Ciofani et al., publisher and licensee Dove Medical Press Ltd).

Nanomaterials are useful platforms for developing bio-medical technologies which requires the materials not to be harmful, in this sense, nanomaterials interactions with biological systems need to be fully understood to minimise negative effects. In vitro and in vivo administrations of nanomaterials are thereby primarily conducted to attain an exhaustive knowledge of the impacts of nanomaterials on biological systems from molecule to behaviour before being handled in clinical treatments. Ciofani et al. initially investigated BNNTs interactions with various living systems during the last few years. One example which appeared recently was an in vitro investigation on the internalization, uptake mechanism, cytocompatibility, and differentation of poly-L-lysine (PLL)-functionalized BNNTs administrated muscle (C2C12) cells [84]. BNNTs were well coated with PLL in mild conditions and then covalently labelled with fluorescent QDs to make BNNTs visible to confocal microscopy. As shown in Figure 19 a, QDs-labelled PLL-BNNTs (red) were strongly internalized inside cells and broadly distributed in cytoplasm (green). No BNNTs in nuclei (blue) were observed probably due to their large size. Inhibitation studies with sodium azide treated cells indicated that the majority of BNNTs were prevented from being taken up and located outside of the cell membrane, only very few tubes were found inside cells (Figure 19 b), suggesting that cells taken up by BNNTs were mainly through ATP associated energy-dependent endocytosis. Cytocompatibility studies via multiple in vitro assays suggested that PLL-functionalized BNNTs had no apparent negative effects on C2C12 cells up to a concentration of 10 μg/mL after 72 hours incubation. Cells treated with PLL-functionalized BNNTs were able to proliferate, differentiate structure, and express protein and DNA to the same level as negative control cells. These investigations provided important information, supporting the idea that PLL-functionalized BNNTs are promising nanovectors. In 2012, Ciofani et al. reported for the first time a pilot toxicological assessment after a single instillation of glycol chitosan-functionalized BNNTs in rabbits at a dose of 1 mg/kg [85]. Assessment of blood contents, liver and kidney functions were carried out after 52 hours following an injection, the results indicated that BNNTs have no negative effects on rabbits at the instillation conditions. Furthermore, in vitro and in vivo experiments are strongly needed to investigate the long-term effects of BNNTs on biological systems. Nonetheless, these investigations strongly support the idea that noncovalently functionalized BNNTs are considerably biocompatible and have negligible negative effects, thus showing great promise for developing various nanobiotechnologies and multifunctional nanoplatforms in biology and medicine.

## 4. Summary and perspective

**Figure 20.**
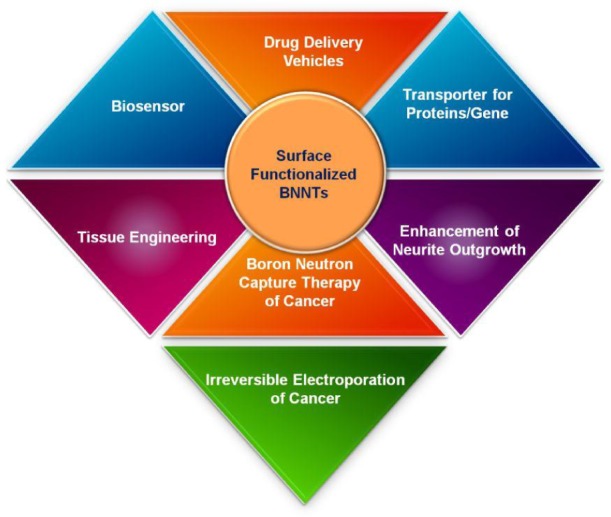
Potential applications of surface functionalized BNNTs in nanobiomedicine

In this article, the latest advancements in the noncovalent functionalization of BNNTs with polymers and biomolecules were outlined. The noncovalent interactions between BNNTs and functional molecules allow for their efficient attachment onto the sidewall of BNNTs, thereby endowing a good dispersity and new chemical functions to BNNTs. Meanwhile, the applications of those functionalized BNNTs for a superhydrophobic surface, visible-light emission, QDs decoration, protein immobilization, length cutting, and evaluation of in vitro/in vivo biocompatibility have been further summarized herein to shine light on possible applications in nanobiomedicine. This article should be able to promote interest in the surface functionalization of BNNTs using polymers and biomolecules to advance their applications in cutting-edge nanobiomedicine crossing a broad spectrum from drug/gene delivery, tissue engineering, cancer therapy, to neurobiology (Figure 20).

Although some essential progress has been achieved, the application of BNNTs is still a long way from practical implementation. Many issues regarding surface functionalization and biomedical applications are still not fully understood so far. For example, the detailed mechanism accounting for the toxicity and biocompatibility of functionalized BNNTs are required to be fully investigated using more in vitro and in vivo experiments to gain insight into the full picture of bio-safety in biological systems. The cell-specific recognition function also must be endowed to BNNTs to specifically target them to receptors of cells to explore applications in cutting-edge nanobiomedicine in the future.

To this end, great advancements have been recently achieved in the development of 2-D layered nanomaterials, such as graphene [86, 87], graphene oxide [88], BN nanosheet [89], and nanoribbons [90]. Perfectly isolated 2-D layered nanomaterials can be considered as just surfacelike materials, because the surface and its edge structures are particularly dominant for the properties of those materials [91]. In other words, the physiochemical properties of these novel nanomaterials are extremely sensitive to surface species and their surroundings; thus, it is important to modify their surfaces and edges [91, 92]. By considering the similarity of the chemical structures, it is possible to functionalize the surface and the edge of those 2-D nanomaterials using various polymers and biomolecules. This should be a new domain for research in bionanoscience and bionanotechnology in the coming years.
